# A Pilot Study of Ketamine Infusion after Suicide Attempt: New Frontiers in Treating Acute Suicidality in a Real-World Medical Setting

**DOI:** 10.3390/ijerph192113792

**Published:** 2022-10-24

**Authors:** Sharvari Shivanekar, Priya Gopalan, Anthony Pizon, Crystal Spotts, Nicolas Cruz, Michael Lightfoot, Rebecca Rohac, Andrew Baumeister, Angela Griffo, Benjamin Panny, Shelly Kucherer, Alex Israel, Manivel Rengasamy, Rebecca Price

**Affiliations:** 1 Department of Psychiatry, University of Pittsburgh, 3811 O’Hara St, Pittsburgh, PA 15213, USA; 2 Department of Emergency Medicine, Division of Medical Toxicology, University of Pittsburgh, 3600 Forbes at Meyran Avenue, Forbes Tower, Suite 10028, Pittsburgh, PA 15213, USA

**Keywords:** suicide, suicidal ideation, suicide attempt, intravenous ketamine, consultation-liaison psychiatry, medical toxicology, tertiary care

## Abstract

Ketamine, in research settings, rapidly reduces suicidal thoughts 2–24 h after a single infusion in patients with high suicidal ideation. In this study, the authors investigate ketamine’s effects on suicidality in a real-world sample of recent suicide attempters on a tertiary-care Consultation-Liaison (CL) psychiatry service. Using an open-label design, 16 transdiagnostic CL patients were recruited, 18–65 years old, to receive a single dose of intravenous ketamine (0.5 mg/kg) in the acute medical setting. All were psychiatrically hospitalized post-infusion. Baseline suicidality and depression measures were compared to ratings taken at 24 h, 5 days, 12 days, and 1, 3 and 6 months post-infusion using paired t-tests. Across all measures, rapid, statistically significant decreases (*p*’s < 0.001) were observed with large to very large effect sizes (Cohen’s *d*’s: 1.7–8.8) at acute timepoints (24 h; 5 days). These gains were uniformly maintained to 6 months post-infusion. Open-label ketamine appeared to rapidly and robustly reduced suicidal symptoms in an ultra-high-risk, heterogeneous, real-world sample. Ketamine infusion may therefore be a safe, feasible, viable method to rapidly reduce suicidality among medically hospitalized patients after a suicide attempt, with potentially enduring benefits. The current pilot findings suggest ketamine could be readily integrated into the settings where high-risk CL patients already receive healthcare, with the potential to become an important and novel tool in the treatment of suicidality.

## 1. Introduction

Suicide is a devastating public health crisis with few available treatments that can quickly reduce the intensity of self-harm thoughts. The World Health Organization (WHO) reports that globally, more than 700,000 people die due to suicide every year. A prior suicide attempt is the single most important risk factor for suicide in the general population [1]. A total of 77% of global suicides occur in low- and middle-income countries. Ingestion of pesticide, hanging and firearms are among the most common methods of suicide globally [2].

Suicide rates in the Unites States remain alarmingly high, increasing by 36% between 2000–2018 and declining by only 5% between 2018–2020. The Center for Disease Control (CDC) reports that in 2020, 1.2 million adults attempted suicide and that there was one death by suicide per 11 min in 2020. Firearms are the most common method used in suicides (52.8%) in the Unites States, followed by suffocation (27.2%) and poisoning (12.0%) [3].

The authors of this study use definitions of suicide, suicidal ideation and behaviors noted by Nock et al. 2008 [4]. *Suicide* is defined as the act of intentionally ending one’s own life. Nonfatal suicidal thoughts and behaviors (hereafter called “suicidal behaviors”) are classified more specifically into three categories: *suicide ideation*, which refers to thoughts of engaging in behavior intended to end one’s life; *suicide plan*, which refers to the formulation of a specific method through which one intends to die; and *suicide attempt*, which refers to the engagement in potentially self-injurious behavior with at least some intent to die [4,5,6,7,8].

Conventional treatments for depression, such as antidepressants, cognitive therapy and electroconvulsive therapy are effective but relatively slow to act, meaning that a substantial number of patients will be left struggling with suicidal thoughts after weeks of treatment. Inpatient hospitalization is the standard of care for patients in suicidal crisis, but as many as 1500 suicides occur during inpatient hospitalization each year [9] and there is additionally a well-known, high-risk period for suicide during the period immediately following discharge from psychiatric inpatient stay [10]. Novel treatment approaches are imperatively needed, particularly given that the incidence rates for suicidal behaviors have remained either unchanged or have increased over recent decades, in spite of increasing access to treatment [4].

Suicidality (i.e., suicidal thoughts and behaviors) is typically treated through a primary focus on treating the DSM diagnoses that confer the risk of these suicidal thoughts and behaviors (e.g., major depression), with less emphasis placed historically on addressing suicide risk directly, across diagnoses [11]. To best address these limitations and bridge the gap between research and clinical practice, novel, rapid-acting treatment approaches and deployment-focused study designs are needed. Intravenous ketamine, an NMDA receptor antagonist used routinely in hospital settings for other indications (e.g., anesthesia, pain management, alcohol withdrawal [12]), shows initial promise as a rapid-acting anti-suicidal medication with the potential for wide dissemination and adoption in inpatient settings. In controlled, outpatient research settings, ketamine rapidly reduces suicidal thoughts as early as 2–24 h after a single infusion in patients with high suicide risk [13,14,15]. Ketamine’s rapid effects on depression and related symptoms (such as suicidality) are often attributed to its ability to rapidly and profoundly reverse neuroplasticity deficits [16,17,18]. Chronic stress leads to sustained decreases in neuroprotective factors (e.g., brain-derived neurotrophic factor (BDNF) expression and signaling) that damage plasticity, fostering neuronal atrophy and synaptic depression, particularly in the PFC and hippocampus [16,17]. Within an integrative conceptual framework that links these deficits to parallel findings in human patients [19], such findings may relate to impairments in experience-based learning, decision making, cognitive flexibility and prefrontal inhibition seen in suicidal patients [20,21,22,23,24,25,26], which, in turn, could produce inflexible negative biases in cognition and behavior, such as rigidly held negative and death-related representations of their self—a key prospective risk factor for suicidality [27,28,29].

Far fewer studies have examined ketamine’s impact on “real-world,” heterogeneous, transdiagnostic psychiatric samples with key comorbidities that confer the risk of poor outcomes (e.g., substance disorders) and who are at a severe, acute risk of suicide, and thus, are often excluded from research protocols. Promising data on the use of intravenous or intranasal ketamine in relatively small samples of such high-acuity patients, delivered in emergency psychiatric treatment settings, provide evidence for the safety and potential efficacy of such an approach [30,31]. Prior work has not fully leveraged an effectiveness or “deployment-focused” model [32], in which research focuses on the kinds of individuals and intervention contexts for which the treatments are ultimately intended.

Emerging research in patients with addiction [33,34], depression [35], post-traumatic stress disorder [36,37] and obsessive-compulsive disorder [38] lend some initial support to a hypothesized synergy between ketamine and existing behavioral learning-based treatment approaches, which might allow for such longer-term change to occur. Due to its acute enhancing impact on neuroplasticity (e.g., synaptogenesis) [19], it has been suggested that ketamine might prime patients to respond to other first-line treatments by widening rigid, maladaptive responses and facilitating the acquisition of new skills and/or cognitive-behavioral patterns [39]; however, this theoretical model has not previously been deployed in the context of “real world” standard care, i.e., through the sequencing of a ketamine infusion prior to standard intensive psychiatric treatment, and through the tracking of longer-term outcomes to assess for both detrimental and beneficial impacts of this approach.

In this pilot, open-label effectiveness study, the authors investigated ketamine’s effects on suicidal thoughts and behaviors in a real-world sample of recent suicide attempters at an imminent risk of suicide on a tertiary-care Consultation-Liaison (CL) psychiatry service. This study was conducted acutely following the suicide attempt and patients were followed up over a six-month period. Ketamine infusion was delivered during inpatient medical stabilization, prior to their transfer to psychiatric inpatient care, in coordination with the CL psychiatry service and relevant emergency medicine physicians and providers on services routinely treating many patients who have attempted suicide (e.g., Medical Toxicology; Trauma Surgery). This represents a novel deployment-focused delivery model aimed at the rapid stabilization of suicidality, with the potential for wide dissemination beyond specialized academic psychiatric settings, given ketamine’s routine and widespread use among medical inpatient providers involved in acute care following a medically serious suicide attempt. Through the administration of a single “pre-treatment” infusion during medical stabilization, delivered by embedded clinicians shortly prior to psychiatric hospital transfer, the authors hypothesized ketamine would (1) rapidly impact suicidal ideation in patients who attempted suicide (from 1 to 12 days following infusion) and (2) confer enduring benefits over a 6-month follow-up, possibly by enhancing the response to standard psychiatric inpatient treatment-as-usual, which included both behavioral and pharmacological therapies administered at the full discretion of inpatient providers (who were not involved in the research study) in a treatment-effectiveness design. In exploratory analyses, patients enrolled in the study were matched on age, gender and diagnosis to a cohort of case–control patients who were seen in the same setting during the same time period, facilitating comparisons of exploratory outcomes based on electronic medical records.

## 2. Materials and Methods

**Figure 1:**
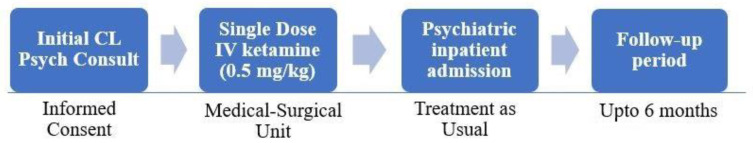
Overview of study design.

*Participants.* All research methods were approved by the University of Pittsburgh Institutional Review Board and pre-registered on clinicaltrials.gov (NCT04154150) (accessed on 20 September 2022). A total of 16 hospitalized adult patients who attempted suicide [mean age = 34.01 (SD = 16); 8 female, 7 male; 1 transgender individual who identifies as male] were enrolled in an open-label ketamine study between December 2019 and January 2021. Patients were initially identified through consults placed to the Consultation-Liaison (CL) psychiatry service during the course of their medical stabilization following suicide attempt, and were further reviewed for initial eligibility criteria through chart reviews and consultations with medical and CL providers (see Figure 1). Inclusion criteria included: aged 18–65; medical inpatients who were referred for psychiatric consultation/liaison due to suicidality and were determined by psychiatric CL to require inpatient psychiatric hospitalization; possessed a level of judgment and understanding sufficient to consent to all procedures required by the protocol; be deemed an appropriate and reasonable medical candidate for intravenous ketamine by a physician authorized to prescribe medication to the patient during inpatient hospitalization. Exclusion criteria included: the presence of current acute psychosis, autism spectrum disorder, or current delirium; the use of a Monoamine Oxidase Inhibitor (MAOI) within the previous 2 weeks; were pregnant or breastfeeding; had a reading level of <5th grade as per the WRAT-3 reading subtest [40]; had a past intolerance or hypersensitivity to ketamine; were currently taking contraindicated medications—St John’s Wort, theophylline, tramadol, or metrizamide; had received ECT in the past 6 months prior to intake; were at risk of withdrawal-related issues (e.g., delirium tremens, severe opiate withdrawal) or had presented with substance-induced psychosis.

Primary diagnoses, as determined by a structured diagnostic (MINI) interview [41], included major depressive disorder (31.3%), major depressive disorder with past psychotic features (12.5%), depressive disorder NOS (12.5%), bipolar I (25%), bipolar I with past psychotic features (6.3%) and bipolar II (12.5%). A total of 19% of the sample (6 patients) had one or more comorbid substance-use disorders.

A total of 16 case–control patients that met the same eligibility criteria (with the exception of reading level, which was not assessed) were identified through CL consults for patients who attempted suicide during the same time period, but were individuals who, due to time and staffing constraints, either were not enrolled by the study team (*n* = 13), who had consented but did not complete any study procedures (*n* = 2) or who declined to participate upon approach (*n* = 1). Case–control patients were matched one-to-one to enrolled participants as closely as possible on gender, age (within 5 years), race and diagnosis (bipolar vs. unipolar depression, as recorded in the EMR), resulting in a control sample with similar demographic and clinical characteristics to the ketamine sample (see Table 1). Case–control patients were not enrolled, and thus, only the outcome measures available through the EMR (see below) were available.

*Assessments: Primary outcomes.* See Figure 1 for a flowchart of study events. All clinical measures that were used have been validated in prior research and exhibit excellent psychometric properties. Clinical assessments were collected at the pre-infusion baseline and at the following post-infusion timepoints: 1, 5 and 12 days and 1, 3 and 6 months. Masters-level clinical raters with extensive training and experience in conducting depression treatment research administered the Montgomery–Asberg Depression Rating Scale (MADRS) [42] to assess total depression severity and severity of SI; the MADRS-SI item (item 10) and the Scale for Suicide Ideation (SSI; [43]; added to the protocol after the first 4 patients) to assess the severity of SI; and the Columbia Suicide Severity Rating Scale (CSSRS; [44]) to capture the most severe form of SI and suicidal behaviors (e.g., attempts) during the interval since the last assessment. As secondary outcomes, patients also completed the Adult Suicide Ideation Scale (ASIQ) [45], a 25-item self-report measure of SI frequency and severity, and the Quick Inventory of Depressive Symptoms—Self-Report (QIDS-SR) [46]. At post-ketamine timepoints, the timeframe captured by clinical raters, as well as the specific response choices provided on the ASIQ, were adjusted as needed to reflect the brief duration of the assessment interval (e.g., 1 day). The retention at post-infusion timepoints was 100% at day 1, 94% at day 5, 63% at day 12, 56% at month 1, 69% at month 3 and 75% at month 6. At the screening, the MINI International Neuropsychiatric Interview [41] was administered to characterize Axis I diagnoses, and the WRAT-3 Reading test [47] was administered to ensure that a minimal 5th-grade reading level was met.

*Exploratory chart review.* Because attrition was anticipated during the follow-up due to the severe degree of illness and multiple comorbidities among the target sample, electronic medical record (EMR) chart reviews were performed on all records within the University of Pittsburgh Medical Center hospital system, which includes 40 hospitals and 800 outpatient sites that serve a regional catchment area covering Pennsylvania, and portions of Ohio, New York and Maryland. Chart review procedures were designed to capture baseline features and relevant outcomes among both enrolled participants and case–controls, and were conducted by trained raters following a structured protocol, under the supervision of the study PI. The following outcome metrics were recorded: length of psychiatric inpatient stay following index attempts (days); total # of inpatient therapy groups attended during the initial inpatient stay; attendance at a first scheduled psychiatric outpatient care visit following hospital discharge (yes/no); subsequent psychiatric, substance-related, and medical inpatient stays during the follow-up period (#, duration); and total # and types of outpatient visits during the follow-up among broad categories (e.g., medication management; psychotherapy; substance-related).

*Infusion procedure.* Infusions of intravenous racemic ketamine (0.5 mg/kg in saline) were prepared by the hospital pharmacy and were administered over 40 min by experienced, embedded, licensed nurses in an inpatient setting, with continuous cardiovascular monitoring and routine checks regarding the maintenance of patient comfort during and after infusion. In the event that cardiovascular readings transgressed pre-specified parameters based on hospital guidelines for subanesthetic ketamine infusion monitoring (e.g., systolic blood pressure ≥ 180 or ≤90 mmHg and a change of ±20 mmHg from baseline; diastolic blood pressure ≥ 105 or ≤50 mmHg and a change of ±20 mmHg from baseline) or other adverse events were noted (e.g., patient distress, confusion, or lack of arousability), the prescribing physician—a medical toxicology physician with extensive clinical experience prescribing intravenous ketamine—was available to provide immediate emergency intervention. CL psychiatrists involved in the patient’s care were additionally on call in the event that further psychiatric management was indicated.

*Additional interventions.* Participants were transferred to standard psychiatric inpatient care within 1–4 days of ketamine infusion (based on bed availability). Inpatient units within the University of Pittsburgh Medical Center’s Western Psychiatric Hospital included several general adult (non-psychotic) inpatient units (*n* = 11 patients in the ketamine sample), as well as a specialized dual diagnosis unit (*n* = 5 patients). In standard adult inpatient units, treatment involved daily group milieu therapy sessions incorporating mindfulness and CBT techniques, medication management, safety planning (documented at discharge) and liaisons with social services as needed. In the dual diagnosis unit, standard care included all of these strategies as well as individualized Motivational Interviewing techniques delivered daily by the unit chief (an expert in MI) and his supervisees.

For 4 days following infusion, patients were also invited to complete a sequence of very brief (20 min), fully automated, computer-based cognitive training sessions, administered by research staff. Patients were randomized to receive either “active” cognitive training (designed to entrain implicit associations between self-relevant and positive content (words and pictures); *n* = 8) or “sham” cognitive training (identical computer exercises with neutral rather than self-relevant or positive content; *n* = 8). Due to low statistical power for comparisons in this pilot study, the two CT groups were pooled together in all analyses and were not considered further in the present manuscript.

## 3. Results

*Adverse events.* Adverse events reported within 1 day of the infusion were mild and largely consistent with the known side effect profile of subanesthetic ketamine (Table 2). One patient experienced moderate anxiety, distress and nausea approximately 20 min into the infusion and requested to discontinue; the infusion was stopped, study physicians were immediately notified and ondansetron was prescribed for nausea. After ketamine discontinuation, the patient’s distress quickly subsided without the need for further intervention. The patient continued in the remainder of the research protocol without further incidents. The patient’s data were included in all reported analyses, in accordance with an intent-to-treat principle. No other infusions required enhanced physician oversight or intervention, based on either pre-specified vital parameters or other adverse events.

**Figure 2:**
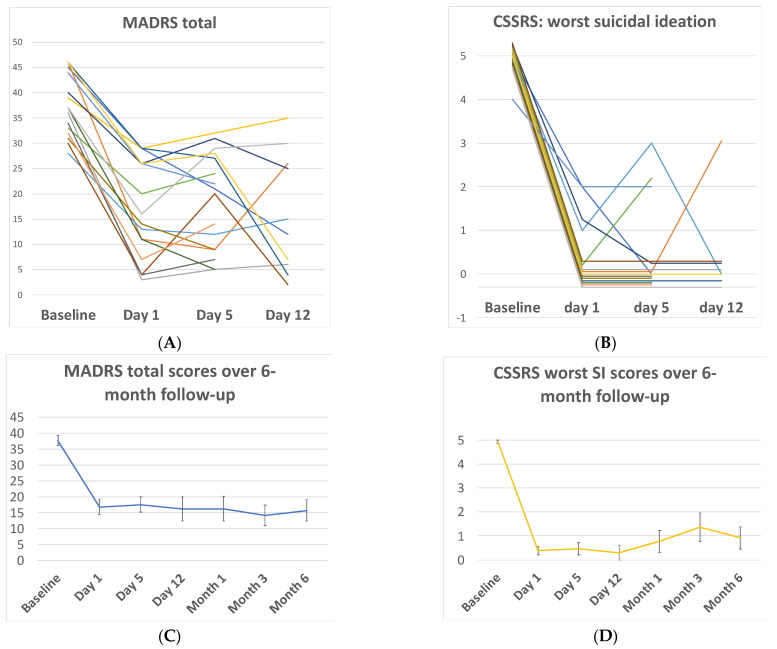
(**A**) Total depression severity scores measured by the Montgomery-Asberg Depression Rating Scale (MADRS) in each individual patient as a function of time during the first 12 days post-enrollment. (**B**) Worst suicidal ideation scores measured by the Columbia Suicide Severity Rating Scale (CSSRS) in each individual patient as a function of time during the first 12 days post-enrollment. (**C**) Group mean total depression scores from baseline to month 6. (**D**) Group mean worst suicide ideation scores from baseline to month 6.

*Rapid outcomes: ketamine sample.* At 1-day post-infusion, total depression and SI scores decreased rapidly from baseline across all measures, with 95% of CIs reflecting large-to-very-large effect sizes (Table 3; Figure 2). At day 1, 56.3% of patients were classified as treatment ‘responders’ based on a ≥50% decrease in the total depression (MADRS) score, and 75% of patients reported full remission of all passive or active SI, per the CSSRS-SI scale (worst SI score = 0). These rapid decreases from baseline on all measures were maintained at both 5 and 12 days post-infusion (Table 3; Figure 2).

*Longer-term outcomes: ketamine sample.* In the ketamine-treated patients completing follow-up interviews and questionnaires at each timepoint, significant mean decreases from the baseline on all measures were continuously maintained at the 1-, 3-, and 6-month post-infusion follow-ups (Table 3; Figure 2), with only one exception (the QIDS-SR, a secondary self-report measure at months 3 and 6).

Consistent with the high-risk sample and the high-risk, post-discharge period for the follow-up, two patients (12.5%) reported a repeated suicide attempt at one or more follow-up assessments, per the CSSRS, and were treated for acute suicidality within our hospital system.

*Exploratory EMR outcomes: ketamine and case–control samples.* When comparing the ketamine-treated sample to case–controls, there were no significant between-group differences in the length of the initial inpatient hospital stay (ketamine: mean = 11.63, SD = 15.76; case–controls: mean = 7.33, SD = 5.42; *t*_29_ = −1.0, *p* = 0.326), the number of therapy groups attended during the inpatient stay (ketamine: mean = 32.63, SD = 59.72; case–controls: mean = 18.86, SD = 12.31; *t*_28_ = −0.85, *p* = 0.405) or the likelihood of attending the first scheduled outpatient visit post-discharge (33% of the ketamine group; 50% of the control group; Fisher’s exact *p* = 0.46). Five patients (15.6%) re-attempted suicide during the follow-up interval. This included three ketamine-treated patients (including two patients who completed at least one follow-up assessment where the SA suicide attempt was captured via clinical interview, and one additional patient for whom the suicide attempt was captured via electronic chart review) and two case–controls; no significant between-group difference: Fisher’s exact *p* = 1.00. Nine patients underwent psychiatric re-hospitalization for recurrent symptoms related to their baseline diagnoses (six ketamine-treated patients and three case–controls; no significant between-group difference: Fisher’s exact *p* = 0.433). Nine patients underwent re-hospitalization for medical conditions during the follow-up interval (three ketamine-treated patients and six case–controls; no significant between-group difference: Fisher’s exact *p* = 0.44). Regarding substance use-related outcomes, two patients (in the case–control group) had a substance-related inpatient or rehab stay recorded during follow-up, one patient in the ketamine group and two patients in the case–control group had an accidental substance use-related overdose, and one patient in each group had a urine test recorded as positive for substance use during the follow-up period. Ketamine and control patients did not differ on the frequency or types of outpatient visits completed during the follow-up (*p*’s ≥ 0.394). No patients completed suicide or died by other causes during the follow-up interval.

## 4. Discussion

### 4.1. Main Findings 

In this pilot effectiveness trial, ketamine infusion was safe, feasible and strongly effective at rapidly reducing depressive symptoms and suicidal thoughts among a high-risk, transdiagnostic sample of recent suicide attempters. In follow-up clinical assessments over a 6-month window, large reductions from baseline were continuously maintained (Table 3; Figure 2). Re-attempt rates in both ketamine-treated and case–control patients were in line with, or lower than, prevalence estimates among attempters for the acute 6-month period following psychiatric inpatient discharge [48,49,50]. Suicide can be conceptualized as a multifactorial phenomenon. The mechanisms of the underlying vulnerability for suicidal behavior are the focus of ongoing research but are thought to include biological, social and psychological underpinnings [51,52]. Ketamine may rapidly address certain key molecular and neurocognitive substrates of suicide risk, potentially through plasticity-enhancing effects and/or other key pro-cognitive and neuroprotective impacts [19]. As such, the results from this study may have broad implications on clinical care, system-based approaches to suicide management and the optimization of scarce resources.

### 4.2. Implications for Clinical Care and Resource Management for Suicidality

The financial toll of suicide on society is high. Suicides and suicide attempts cost the United States over USD 70 billion per year in lifetime medical and work-loss costs alone [53]. Furthermore, the COVID-19 pandemic has increased suicide risk in individuals with preexisting mental health issues, as they are likely to be affected by illness relapse, interruption in psychiatric services, increased isolation and possible exacerbation by anxiety due to the pandemic [54]. Concurrent to these public health considerations, access to psychiatric beds is a topic of national urgency with grim reports on the boarding of psychiatric patients in emergency departments (EDs) [55]. In Kraft et al.’s study of mental health patients with prolonged ED stays, the primary barrier to disposition was the lack of patient acceptance to inpatient psychiatric hospitals, community settings or other housing [56]. ED length of stay (LOS) is associated with increased ED crowding, increased costs, decreased quality of care, decreased staff morale and decreased patient satisfaction. The longest LOS has been found to be for patients who were suicidal or inflicting self-harm, higher than schizophrenia, other psychotic and mood disorders [57]. As a rapidly acting intervention for acute suicidality, ketamine may become instrumental in decreasing the length of stays in ED and inpatient psychiatry settings. Data from this study suggest that ketamine administration in real-world inpatient medical settings in the immediate aftermath of a serious suicide attempt is both feasible and safe, and may be effective even across a broad transdiagnostic range of suicide attempters with multiple comorbidities and severe illness.

Clinical care implications for rapidly acting antidepressants are broad and include predominantly patients with mood disorders with suicidal thoughts. Additional populations that require consideration include those with parasuicidal behaviors, including ingestions and self-injurious behaviors, such as cutting. As repeated psychiatric hospitalizations may be detrimental for the care of these patients, they may be particularly benefitted by a rapidly acting intervention that can be administered in the medical setting [58,59].

Results from this study could also be extended to other medically hospitalized populations for whom rapid antidepressant effects could be invaluable. Examples include in palliative care settings, where end-of-life time considerations may preclude long treatment trials [60], or in dementia care [61]. For patients with substance-use disorders (SUDs), including those who are critically ill [62], rapid antidepressant effects may allow for better engagement with post-discharge care [33,34].

### 4.3. Comparison to Other Studies of Rapidly Acting Antidepressant and Anti-Suicide Agents

Several newer biological agents are under investigation to treat severe depression and suicidality. The time-limited, short-term use of very low dosages of sublingual buprenorphine was associated with decreased suicidal ideation in severely suicidal patients without substance abuse [63]. Brexanolone [64,65] and zuranolone [66] are two novel antidepressant agents that are being studied in the use of postpartum depression. These rapid-acting agents are allopregnanolone analogues that seek to physiologically replicate changes to the postpartum hormonal milieu. Brexanolone is intravenously administered as a 60-h infusion and is approved by the Food and Drug Administration (FDA), but has not been studied explicitly for suicidality. Zuranolone is an oral medication currently being studied for postpartum syndromes but studies have extended to major depressive disorder [67], Parkinson’s disorder [68], and sleep disorders [69]. While they act more rapidly than traditional “anti-suicide agents”, such as lithium and clozapine, these more recent therapies are still limited by the time lag between administration and improvement in suicidal thinking.

The Oral Ketamine Trial on Suicidality (OKTOS) is an open-label trial of sub-anesthetic doses of oral ketamine over 6 weeks. Six weeks of oral ketamine treatment in participants with chronic suicidality led to a significant reduction in suicidal ideation [70]. The authors reported that the response observed in this study is consistent with IV ketamine trials; however, the study excluded patients with acute suicidality, history of psychosis, mania/hypomania and several medical conditions, including abnormal liver function testing, history of stroke, cardiovascular disease and hypertension.

Similarly, other prior studies of ketamine treatment for suicidality have been limited by focusing on illustrating its rapid, short-term effects, while lacking longer-term follow-ups. Such data are needed in order to allay fears that have been raised of potential iatrogenic effects (e.g., premature discharge from the inpatient stay; increased substance use, including ketamine abuse) [71,72] and to understand whether ketamine’s rapid effects alter longer-term trajectories and behaviors in a meaningful way (e.g., reducing suicide re-attempts among high-risk samples during key windows of risk, such as during 6 months post-discharge from inpatient hospital stay) [48,49,50]. Philips et al. in a 2020 study compared the reduction in SI elicited by a single ketamine infusion with an active control, and found that repeated doses prolonged the suppression of SI. However, this study excluded those with substance-use disorders and BMI > 35 [73].

This pilot study provides initial support for the feasibility and safety of a single dose of ketamine to rapidly reduce both depression and suicidal ideation, with reductions in research assessments sustained over a six-month period (Figure 2, Table 3) when provided in the context of subsequent, standard inpatient psychiatric care. Importantly, those with substance-use disorders, which are highly prevalent among individuals attempting suicide and confer a high risk of poor long-term prognosis, were included in this sample (Table 1). Substance-use disorders significantly increase the risk of suicide attempts [74,75,76]; indeed, up to 40% of patients seeking treatment for substance dependence report a history of suicide attempts [77], thus, highlighting the significance of including this cohort of patients in the current study. Abbar et al. used a single dose of IV ketamine for suicidality in a 2022 double-blind, randomized control trial (RCT) named KETIS [78]. This study has several similarities to the present study, as well as notable strengths including the RCT design and large sample size (*n* = 156); however, patients with substance-use disorders, psychosis, and those who were committed involuntarily for psychiatric treatment were excluded. In an effort to measure the impacts of ketamine in a highly generalizable and fully unbiased sample, the current study was inclusive of a much broader range of patients, including those with a history of substance use, psychosis and a current involuntary admission status.

In exploratory analyses using electronic chart-based indices over the 6-month follow-up, no significant differences emerged between this recruited sample and a matched case–control sample of hospitalized suicide attempters. The overall consistency of chart-based outcomes may be viewed as providing initial reassurance with respect to the safety and longer-term impact of ketamine infusion among patients traditionally excluded from randomized controlled trials of ketamine and other agents (e.g., undergoing an acute suicidal crisis, comorbid substance-use disorders, history of psychosis or mania). Due to the use of a deployment-focused, effectiveness design with minimal exclusionary criteria, these results could have wide-reaching relevance to a range of clinical settings in which suicide attempters are seen, and, with further replication and confirmation of findings, could support ketamine’s strong potential for dissemination in a diverse range of healthcare settings.

### 4.4. Patient Perspective

The authors would be remiss not to mention the perspectives of the participants of this study, who consistently expressed a hope that their participation would help not only them, but others suffering with depression and suicidality in the future. The authors express gratitude to these stoic individuals who chose to participate in this study amid a painful and chaotic time in their lives.

### 4.5. Limitations

The current study was limited by its open-label design and small sample size, which increases the risk of statistical errors and does not allow for differentiation between ketamine-specific effects and non-specific effects, such as the impact of inpatient treatment. More specifically, almost all of the patients were on at least one psychotropic medication at the time of the ketamine infusion, and as such, it is not possible to separate ketamine’s own effects from any potential synergy with other psychotropics and any benefits from the inpatient psychiatric admission and treatment-as-usual that followed the ketamine infusion. However, regarding the rapid onset of effects observed at 24 h, it is worth noting that these assessments took place during the medical stabilization stage, prior to the onset of any specialized psychiatric care. We were not able to obtain a complete consistent follow-up assessment for all 16 patients for the entire six-month follow-up period due to difficulties in contacting patients post-discharge, which could introduce bias into the reported findings.

## 5. Conclusions

A single subanesthetic dose of IV ketamine, administered in the medical setting during routine stabilization following a suicide attempt may reduce the intensity of depression and suicidal ideation in a rapid and sustained manner. Several limitations of this pilot study will be addressed in the authors’ ongoing randomized controlled trial (R01MH124983; clinicaltrials.gov: NCT04578938) (accessed on 20 September 2022), where the aim is to recruit 200 patients who are admitted to a medical hospital following a suicide attempt and are randomized to receive either ketamine infusion (*n* = 100) or no infusion (treatment-as-usual; *n* = 100). Patients will receive structured scales to measure suicidality and depression throughout a 12-month follow-up period. Based on the current pilot study findings, further studies of ketamine’s feasibility, safety and therapeutic potential are warranted utilizing a real-world, deployment-focused approach to reach patients at the highest need, in the settings where they already receive care.

## Figures and Tables

**Table 1:** Baseline demographic and clinical characteristics of the ketamine-treated sample and the case–control sample.

	Ketamine-Treated Sample (*n* = 16)		Case-Control Sample (*n* = 16)	
Race, *n* (%)				
White	13	(81.3%)	13	(81.3%)
Black	3	(18.8%)	2	(12.5%)
Unknown		--	1	(6.3%)
Gender, *n* (%)				
Female	8	(50%)	8	(50%)
Male	7	(43.8%)	7	(43.8%)
Transgender (female-to-male)	1	(6.3%)	1	(6.3%)
Age, mean (SD)	34.06	(16.06)	35.56	(16.46)
Taking psychotropic medication at time of index SA, *n* (%)	15	(93.8%)	14	(87.5%)
Number of psychotropic medications, mean (SD)	2.67	(1.59)	2.64	(1.39)
Principal diagnoses, *n* (%) ^¥^				
Unipolar				
Major Depressive Disorder	5	(31.3%)	2	(12.5%)
Major Depressive Disorder w/Psychotic Features	2	(12.5%)	0	(0%)
Depressive Disorder NOS	2	(12.5%)	7	(43.8%)
Bipolar				
Bipolar I Disorder	4	(25%)	2	(12.5%)
Bipolar I Disorder w/Psychotic Features	1	(6.3%)	1	(6.3%)
Bipolar II Disorder	2	(12.5%)	2	(12.5%)
Other Bipolar and Related	0	(0%)	2	(12.5%)
Comorbid alcohol- or substance-use disorder, *n* (%)	5	(31.3%)	2	(12.5%)

Note: No variables in the table above differed as a function of group (ketamine-treated vs. case–control patients) according to unpaired t-tests (for continuous variables) or Chi-squared tests (for categorical variables) (*p*’s > 0.19). ^¥^ Established by the Mini International Neuropsychiatric Interview for ketamine patients and the Electronic Medical Record review for case–control patients; this led to an increased prevalence of non-specific diagnoses (Depressive Disorder NOS; Other Bipolar and Related) in case–control patients who were not given a structured interview to clarify diagnosis. Groups were matched on the presence of unipolar vs. bipolar forms of depression at baseline.

**Table 2:** Adverse events: symptoms reported as new or worsening from baseline during the infusion and/or within the subsequent 24 h period.

Symptom	Frequency (*n*)	% (of 16 Patients)
Anxiety	5	31.25
Constipation	4	25.00
Decreased energy	3	18.75
Diarrhea	1	6.25
Difficulty sleeping: too little	6	37.50
Difficulty sleeping: too much	1	6.25
Dizziness	4	25.00
Dry mouth	2	12.50
Emotional indifference	5	31.25
Feeling ‘high’	1	6.25
Headache	5	31.25
Increased appetite	3	18.75
Loss of sexual desire	1	6.25
Nausea	3	18.75
Restlessness	3	18.75
Sweating	5	31.25
Tremors	1	6.25
Trouble achieving orgasm	1	6.25

**Table 3:** Paired t-test results and effect sizes (Cohen’s d) for continuous measures of depression and suicidal ideation, relative to baseline, within the ketamine-treated sample (*n* = 16).

Measure	Baseline (*n* = 16)	Day 1	Day 1 Statistics	Day 5	Day 5 Statistics	Day 12	Day 12 Statistics	Month 1	Month 1 Statistics	Month 3	Month 3 Statistics	Month 6	Month 6 Statistics
MADRS total	37.75 (6.2)	16.75 (9.71)	*n* = 16, *t*_15_ *=* 11.3, *p* < 0.001, *d* = 2.6 [1.6,3.4]	17.53 (9.28)	*n* = 15, *t*_14_ *=* 8.7, *p* < 0.001, *d* = 2.5 [1.5,3.4]	16.20 (11.91)	*n* = 10, *t*_9_ *=* 5.5, *p* < 0.001, *d* = 2.4 [1.2,3.4]	16.22 (11.72)	*n* = 9, *t*_8_ *=* 6.6, *p* < 0.001, *d* = 2.3 [1.0,3.3]	14.09 (10.90)	*n* = 11, *t*_10_ *=* 8.8, *p* < 0.001, *d* = 2.8 [1.5,3.8]	15.67 (11.68)	*n* = 12, *t*_11_ *=* 7.0, *p* < 0.001, *d* = 2.4 [1.2,3.3]
MADRS-SI	5.94 (0.25)	0.81 (1.22)	*n* = 16, *t*_15_ *=* 17.0, *p* < 0.001, *d* = 5.8 [4.1,7.3]	0.80 (1.32)	*n* = 15, *t*_14_ *=* 15.3, *p* < 0.001, *d* = 5.4 [3.7,6.8]	0.50 (1.27)	*n* = 10, *t*_9_ *=* 13.7, *p* < 0.001, *d* = 6.1 [3.9,7.9]	1.11 (1.76)	*n* = 9, *t*_8_ *=* 8.3, *p* < 0.001, *d* = 3.9 [2.2,5.3]	1.18 (1.83)	*n* = 11, *t_1_*_0_ *=* 8.7, *p* < 0.001, *d* = 3.7 [2.2,4.9]	1.08 (1.68)	*n* = 12, *t*_11_ *=* 10.2, *p* < 0.001, *d* = 4.0 [2.5,5.3]
CSSRS-SI	4.94 (0.25)	0.38 (0.72)	*n* = 16, *t*_15_*=* 20.5, *p* < 0.001, *d* = 8.5 [6.1,10.4]	0.47 (0.99)	*n* = 15, *t*_14_ *=* 15.4, *p* < 0.001, *d* = 6.2 [4.3,7.7]	0.30 (0.95)	*n* = 10, *t*_9_ *=* 15.7, *p* < 0.001, *d* = 7.0 [4.5,9.0]	0.78 (1.39)	*n* = 9, *t*_8_ *=* 9.1, *p* < 0.001, *d* = 4.3 [2.5,5.7]	1.36 (1.96)	*n* = 11, *t*_10_ *=* 6.0, *p* < 0.001, *d* = 2.4 [1.3,3.5]	0.92 (1.62)	*n* = 12, *t*_11_ *=* 8.7, *p* < 0.001, *d* = 3.6 [2.2,4.7]
ASIQ	91.93 (32.29)	38.20 (30.00)	*n* = 15, *t*_14_ *=* 6.9, *p* < 0.001, *d* = 1.7 [0.9,2.5]	23.07 (8.46)	*n* = 14, *t*_13_ *=* 8.2, *p* < 0.001, *d* = 2.8 [1.7,3.8]	27.17 (27.17)	*n* = 12, *t*_11_ *=* 5.9, *p* < 0.001, *d* = 2.0 [0.9,2.8]	36.90 (38.43)	*n* = 10, *t*_9_ *=* 4.8, *p* = 0.001, *d* = 1.6 [0.5,2.5]	35.10 (37.79)	*n* = 10, *t*_9_*=* 4.8, *p* = 0.001, *d* = 1.7 [0.6,2.6]	43.00 (49.86)	*n* = 7, *t*_6_ *=* 4.1, *p* = 0.006, *d* = 1.0 [−0.2,2.1] ^¥^
SSI	29.29 (1.50)	3.00 (6.27)	*n* = 7, *t*_6_ *=* 9.6, *p* < 0.001, *d* = 5.8 [3.1,7.6]	4.86 (8.25)	*n* = 7, *t*_6_ *=* 7.7, *p* < 0.001, *d* = 4.1 [2.1,5.6]	4.50 (9.00)	*n* = 4, *t*_3_ *=* 5.1, *p* = 0.014, *d* = 3.8 [1.2,5.4]	6.75 (7.23)	*n* = 4, *t*_3_ *=* 6.9, *p* = 0.006, *d* = 4.3 [1.4,6.0]	6.38 (8.70)	*n* = 8, *t*_7_ *=* 7.3, *p* < 0.001, *d* = 3.6 [1.9,5.0]	7.50 (10.56)	*n* = 10, *t*_9_ *=* 6.6, *p* < 0.001, *d* = 2.9 [1.6,4.1]
QIDS-SR	15.9 (5.1)	11.9 (10.6)	*n* = 14, *t*_13_ *=* 2.8, *p* = 0.015, *d* = 0.76 [−0.03,1.5] ^¥^	8.15 (4.76)	*n* = 13, *t*_12_ *=* 4.3, *p* < 0.001, *d* = 1.49 [0.6,2.3]	7.55 (6.2)	*n* = 11, *t*_10_ *=* 4.1, *p* = 0.002, *d* = 1.47 [0.5,2.3]	9.00 (5.68)	*n* = 9, *t*_8_ *=* 3.2, *p* = 0.013, *d* = 1.20 [0.2,2.1]	11.56 (6.40)	*n* = 9, *t*_8_ *=* 2.2, *p* = 0.06, *d* = 0.70 [−0.3,1.6]	12.14 (3.74)	*n* = 7, *t*_6_ *=* 1.19, *p* = 0.28, *d* = 0.48 [−0.6,1.5]

Note: values presented as mean (SD). Paired t-tests compared each post-infusion timepoint to baseline values among the subset of participants who provided data for each measure/timepoint. Cohen’s *d* effect size was calculated as the standardized mean difference (unadjusted for paired comparisons) with 95% confidence interval [,] for Cohen’s d. Variable n’s for specific measures, as listed in the ‘Day/Month X Statistics’ column, are due to partial-visit completion due to time constraints or participant non-compliance. MADRS = Montgomery–Asberg Depression Rating Scale; SI = suicidal ideation; CSSRS = Columbia Suicide Severity Rating Scale; ASIQ = Adult Suicide Ideation Questionnaire; SSI = Scale for Suicide Ideation; QIDS-SR = Quick Inventory of Depressive Symptoms—Self-Report. The ^¥^ 95% CI crosses 0 because a conservative equation for Cohen’s *d* was used which (unlike the corresponding paired t-test) does not adjust for paired comparisons.

## Data Availability

Original data are available to researchers upon request. Please direct inquiries to the corresponding author.
